# Crystal structure, Hirshfeld surface analysis and DFT studies of (*E*)-1-(4-bromo­phen­yl)-3-(3-fluoro­phen­yl)prop-2-en-1-one

**DOI:** 10.1107/S2056989018017371

**Published:** 2019-01-01

**Authors:** Muhamad Fikri Zaini, Ibrahim Abdul Razak, Mohamad Zahid Anis, Suhana Arshad

**Affiliations:** a X-ray Crystallography Unit, School of Physics, Universiti Sains Malaysia, 11800 USM, Penang, Malaysia

**Keywords:** halogen chalcone, crystal structure, DFT, Hirshfeld surface, UV–vis, HOMO–LUMO, mol­ecular electrostatic potential

## Abstract

The asymmetric unit of the title halogenated chalcone derivative, C_15_H_10_BrFO, contains two independent mol­ecules, both adopting an *s-cis* configuration with respect to the C=O and C=C bonds. In the crystal, centrosymmetrically related mol­ecules are linked into dimers *via* inter­molecular hydrogen bonds, forming rings with *R*_1_^2^(6), *R*_2_^2^(10) and *R*_2_^2^(14) graph-set motifs. The dimers are further connected by C—H⋯O inter­actions into chains parallel to [001]. A Hirshfeld surface analysis suggests that the most significant contribution to the crystal packing is by H⋯H contacts (26.3%). Calculations performed on the optimized structure obtained using density functional theory (DFT) at B3LYP with the 6–311 G++(d,p) basis set reveal that the HOMO–LUMO energy gap is 4.12 eV, indicating the suitability of this crystal for optoelectronic and biological applications. The nucleophilic and electrophilic binding site regions are elucidated using the mol­ecular electrostatic potential (MEP).

## Abstract 2

The title halogenated organic chalcone was prepared by a Claisen–Schmidt condensation reaction. A Hirshfeld surface analysis was carried out to reveal the percentage contributions of the inter­molecular inter­actions. A theoretical study was performed using the density functional theory (DFT) at B3LYP with the 6–311 G++(d,p) basis set level to compare with the experimental results of the X-ray analysis and UV–vis absorption analysis in term of the geometrical parameters, HOMO-LUMO energy gap and charge distributions.

## Chemical context   

Chalcones are natural or synthetic compounds belonging to the flavonoid family (Di Carlo *et al.*, 1999 ▸), consisting of open-chain flavonoids in which the aromatic rings are linked by a three-carbon α,β-unsaturated carbonyl system (Thanigaimani *et al.*, 2015 ▸). Chalcone derivatives have attracted significant inter­est in the field of non-linear optics due to their excellent blue-light transmittance, good crystal stability, large non-linear optical coefficients and relatively short cut-off wavelengths of transmittance (Goto *et al.*, 1991 ▸; Patil *et al.*, 2006*a* ▸,*b* ▸; Zhao *et al.*, 2000 ▸). The presence of halogen substitutions results in alterations of the physicochemical properties and biological activities of organic compounds, without introducing much major steric change. As a result of this, many researchers have worked intensively on fluorine substitution to develop a wide range of biologically active materials (O’Hagan *et al.*, 2008 ▸). As part of our studies in this area, fluoro and bromo substituents were introduced in the title compound and the resulting organic mol­ecular crystal is reported herein in term of its structural stability, the percentage contributions of the various inter­actions to the crystal packing, and electronic charge transfer within the mol­ecule.

## Structural commentary   

**Figure 1:**
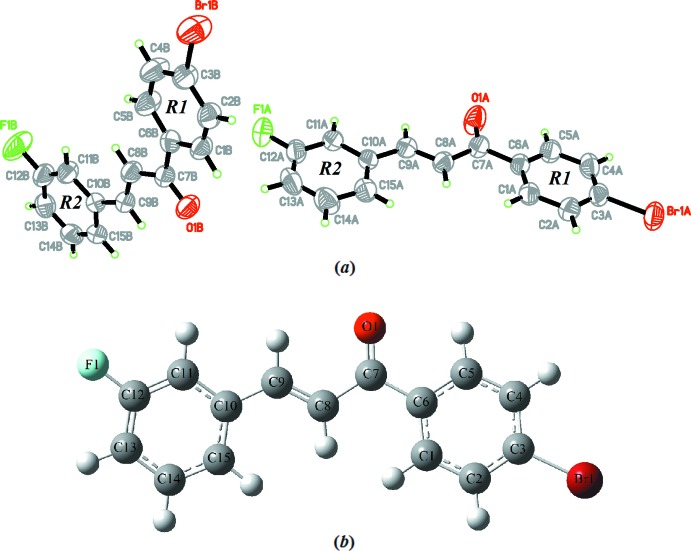
(*a*) The mol­ecular structure of the title compound with displacement ellipsoids drawn at the 50% probability level and (*b*) the optimized mol­ecular structure of the title compound generated using the DFT method at the B3LYP/6–311 G++(d,p) level.

The asymmetric unit of the title compound [Fig. 1 ▸(*a*)] contains two independent mol­ecules (*A* and *B*) with different conformations: the fluoro­benzene group in mol­ecule *A* is rotated by approximately 180° about the C9—C10 bond with respect to mol­ecule *B*, the C9⋯C11—C12—F1 torsion angle formed by non-bonded atoms being 178.4 (3) and −177.0 (3)° in mol­ecules *A* and *B*, respectively. The optimized structure of the title compound was performed with the *Gaussian 09W* software package (Frisch *et al.*, 2009 ▸) using the DFT method at the B3LYP/6-311 G++(d,p) level to provide information about the mol­ecular geometry.
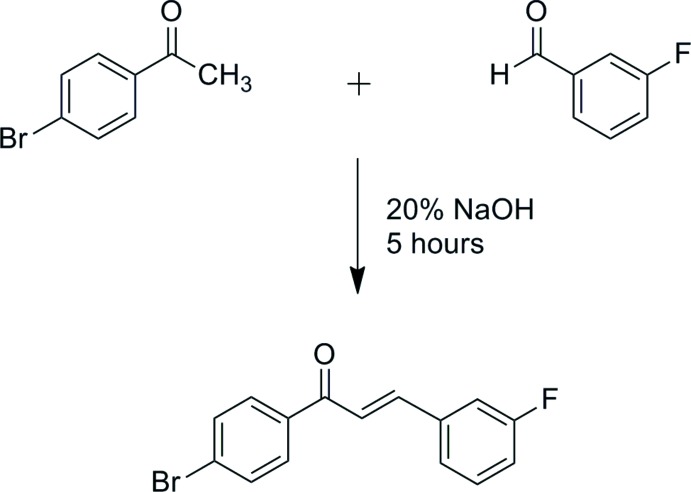


Bond length and angles are unexceptional and fall within the expected ranges. The enone group (O1/C7–C9) of both mol­ecules *A* and *B* adopts *s-cis* configurations with respect to the C7=O1 [C7*A*—O1*A* = 1.207 (4) Å; C7*B-*–O1*B* = 1.221 (3) Å] and C8=C9 [C8*A*—C9*A* = 1.321 (4) Å; C8*B*—C9*B* = 1.322 (4) Å] double bonds. The values of the bond lengths within the enone group obtained by theoretical study are in good agreement with the results of the X-ray analysis (C7–O1 = 1.223 Å; C8–C9 = 1.345 Å). The mol­ecule is essentially planar, the O1—C7—C8—C9 torsion angle being 1.0 (5)° for mol­ecule *A* and 3.9 (4)° for mol­ecule *B*. The corresponding torsion angle from the DFT study is −5.024°. This slight deviation from the experimental value is due to the fact that the optimization is performed in isolated conditions, whereas the crystal environment and hydrogen-bonding inter­actions affect the results of the X-ray structure (Zainuri *et al.*, 2017 ▸). The C1–C6 (*R*1) and C10–C15 (*R*2) phenyl rings in both mol­ecules are approximately coplanar, the dihedral angle they form being 3.75 (15)° and 5.56 (15)° in mol­ecules *A* and *B*, respectively. Furthermore, the dihedral angles formed by the mean plane through the enone group [maximum deviation of 0.004 (3) Å for atoms C7*A*/C8*A*, and 0.016 (3) Å for atom C7*B*] and the *R*1 and *R*2 rings are 6.3 (2) and 2.6 (2)° in mol­ecule *A*, and 6.42 (19) and 4.41 (19)° in mol­ecule *B*.

## Supra­molecular features   

**Figure 2:**
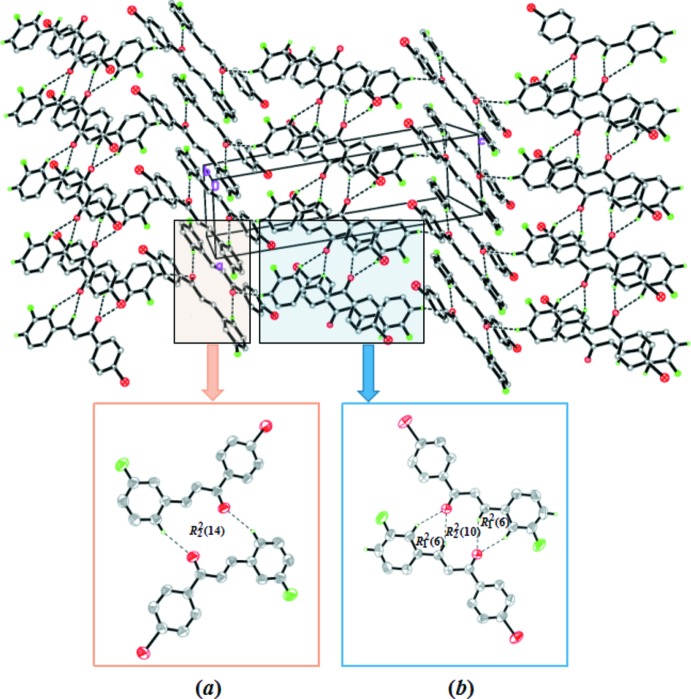
Crystal packing of the title compound showing C—H⋯O hydrogen bonds (dotted lines). H atoms not involved in hydrogen bonding are omitted. The insets show the formation of (*a*) 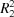
(14) ring motifs and (*b*) 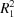
(6) and 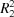
(10) ring motifs.

**Figure 3:**
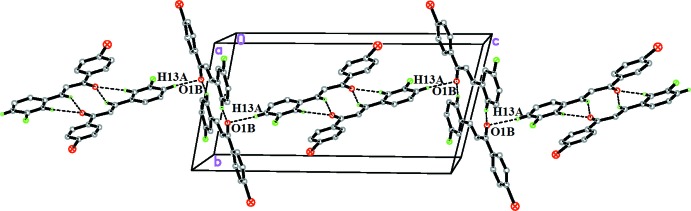
Partial crystal packing of the title compound viewed approximately down the *a* axis showing the formation of a mol­ecular chain parallel to the *c* axis by C—H⋯O inter­actions (dotted lines).

In the crystal packing of the compound, the *B* mol­ecules are centrosymmetrically connected via inter­molecular C15*B*—H15*B*⋯O1*B* inter­actions, forming a ring with an 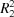
(14) graph-set motif [Table 1 ▸, Fig. 2 ▸(*a*)]. Similarly, the inter­molecular C9*A*—H9*AA*⋯O1*A* and C11*A*—H11*A*⋯O1*A* [Table 1 ▸, Fig. 2 ▸(*b*)] hydrogen bonds also connect the *A* mol­ecules into inversion dimers, forming two 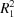
(6) and one 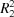
(10) ring motifs. Finally, the C13*A*—H13*A*⋯O1*B* inter­actions act as a bridge, linking the dimers into chains extending parallel to the *c* axis (Fig. 3 ▸).

## Hirshfeld Surface analysis   

**Figure 4:**
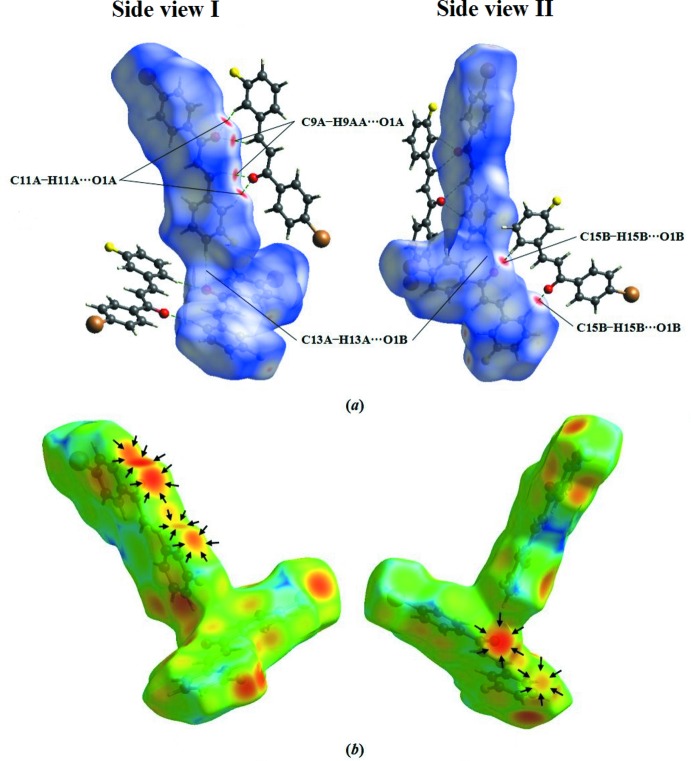
Hirshfeld surfaces of the title compound mapped over *d_norm_* and *d_e_*.

**Figure 5:**
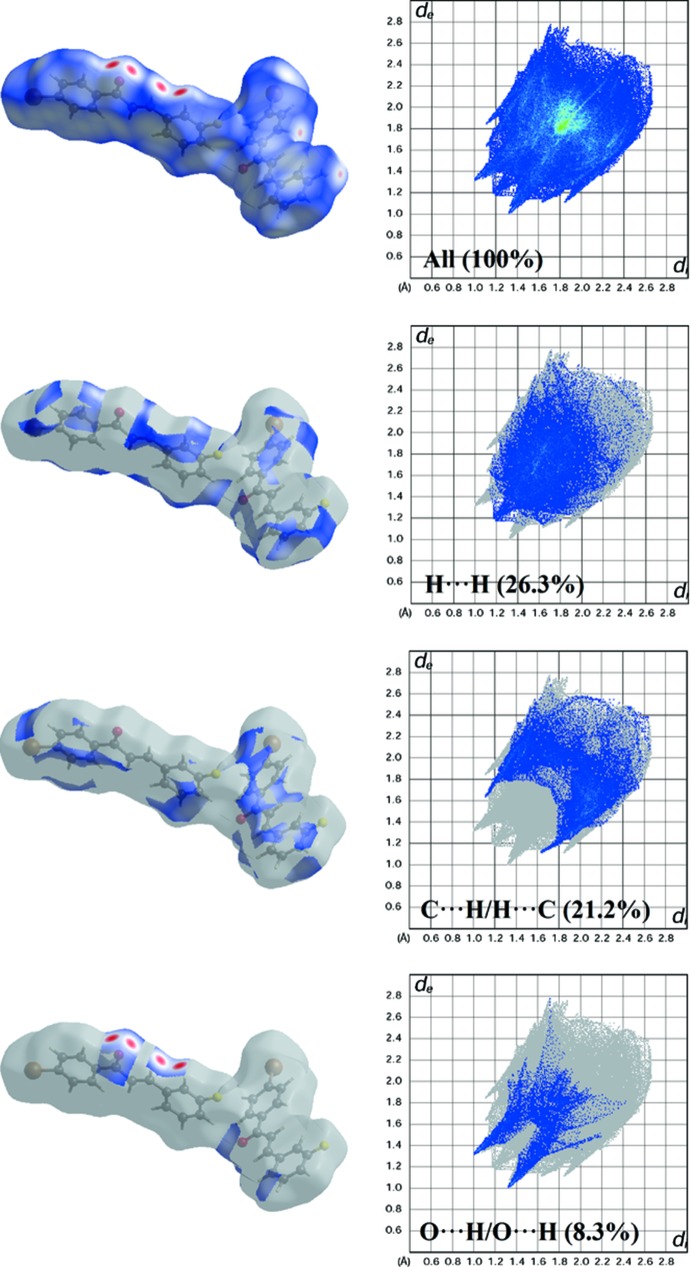
Two-dimensional fingerprint plots with a *d*_norm_ view showing the percentage contributions to the total Hirshfeld surface.

Hirshfeld surface analysis provides the percentage contribution of the inter­molecular inter­actions inside the unit-cell packing. The surface and the related two-dimensional fingerprint plots were generated with *CrystalExplorer3.1* (Wolff *et al.*, 2012 ▸). The *d_norm_* and *d_e_* surfaces are presented in Fig. 4 ▸(*a*) and Fig. 4 ▸(*b*), respectively. All C—H⋯O contacts are recognized in the *d_norm_* mapped surface as deep-red depression areas showing the inter­action between the neighbouring mol­ecules [Fig. 4 ▸(*a*)]. Further existence of these contacts can be visualized under the *d_e_* surfaces. The side view I (Fig. 4 ▸) shows that the *A* mol­ecules may inter­act through C9*A*—H9*AA*⋯O1*A* and C11*A*—H11*A*⋯O1*A* inter­actions, resulting in the formation of three ring motifs. Meanwhile, side view II (Fig. 4 ▸) indicates that for *B* mol­ecules only one ring motif is achieved through C15*B*—H15*B*⋯O1*B* inter­actions. Two-dimensional fingerprint plots provide information about the major and minor percentage contribution of inter­atomic contacts in the compound. The blue colour refers to the frequency of occurrence of the (*d_i_*, *d_e_*) pair and the grey colour is the outline of the full fingerprint (Ternavisk *et al.*, 2014 ▸). The fingerprint plots (Fig. 5 ▸) show that the H⋯H contacts clearly make the most significant contribution to the Hirshfeld surface (26.3%): there is one distinct spike with a *d_e_* + *d_i_* value approximately less than the sum of Van der Waals radii (2.4 Å). In addition, C⋯H/H⋯C and O⋯H/H⋯O contacts contribute 21.2% and 8.3%, respectively, to the Hirshfeld surface. In particular, the O⋯H/H⋯O contacts indicate the presence of inter­molecular C—H⋯O inter­actions where the distance is shorter than the sum of *d_e_* + *d_i_* (∼2.32 Å).

## Frontier mol­ecular orbital and UV–vis Analyses   

**Figure 6:**
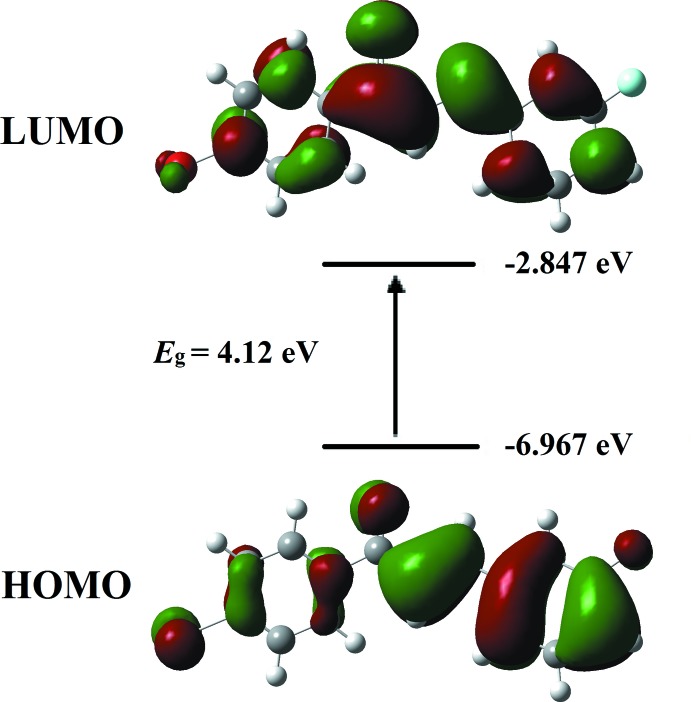
Mol­ecular orbitals showing the HOMO–LUMO electronic transitions in the title compound.

**Figure 7:**
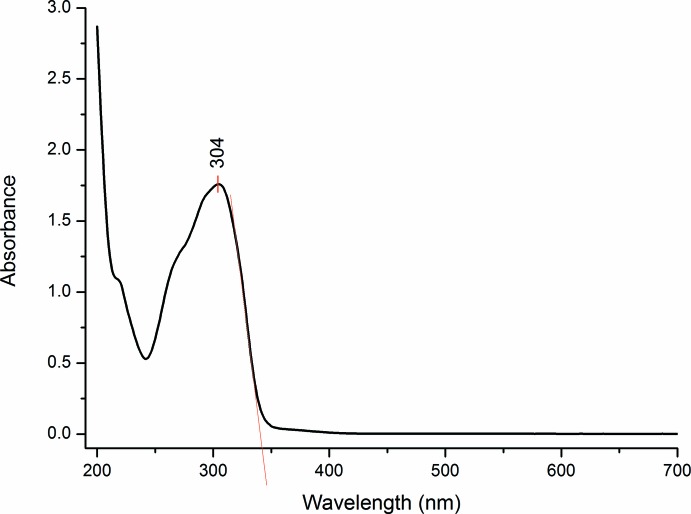
The UV–vis absorption spectrum of the title compound.

Frontier mol­ecular orbital analysis is an important tool in quantum chemistry for studying the mol­ecular electronic charge mobility from the highest occupied mol­ecular orbital (HOMO) and the lowest unoccupied mol­ecular orbital (LUMO). The HOMO–LUMO separation confirms the energy gap of the compound where it is responsible for the ICT (intra­molecular charge transfer) from the end-capping electron-donor groups to the efficient electron-acceptor groups through the π-conjugated path. The electron-density plots of the HOMO and LUMO for the title compound were calculated using density functional theory (DFT) at the B3LYP/6–311 G++(d,p) level. As seen from the orbital plots (Fig. 6 ▸), both HOMO and LUMO extend mainly over the entire mol­ecule, but the mol­ecular orbital localization differs. This can be seen specifically at the enone moiety where the orbital accumulates around the carbon–carbon double bond at the HOMO state whereas it is localized at the carbon–carbon single bond at the LUMO state, indicating conjugation within the mol­ecule. The calculated energy gap, *E*_LUMO_ – *E*_HOMO_, is 4.12 eV. The experimental UV–vis absorption spectrum consists of one major band (Fig. 7 ▸) occurring in the visible region at 304 nm which was assigned to the π–π* transition. This sharp peak was expected to arise from the carbonyl group of the chalcone (Zainuri *et al.*, 2018 ▸). From the UV–vis absorption edge, the calculated energy band-gap value is 3.60 eV, which is similar to that found in a previous study of a related chalcone (Zaini *et al.*, 2018 ▸).

## Mol­ecular electrostatic potential   

**Figure 8:**
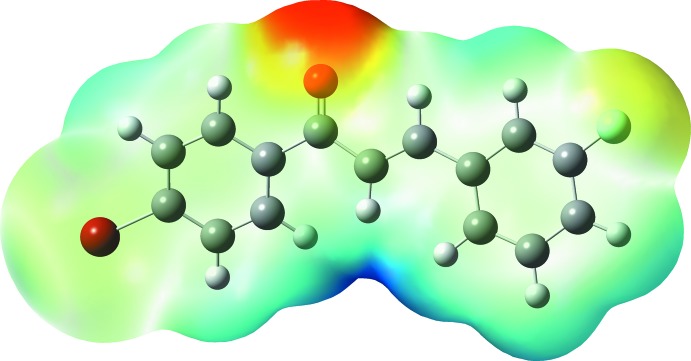
The mol­ecular electrostatic potential surface of the title compound calculated at the DFT/B3LYP/6–311 G++(d,p) level.

The mol­ecular electrostatic potential (MEP) is useful in depicting the mol­ecular size and shape as well as in visualizing the charge distributions of mol­ecules. The MEP map (Fig. 8 ▸) of the title compounds was calculated theoretically at the DFT/B3LYP/6–311 G++(d,p) level of theory. The colour grading in the plot represents the electrostatic potential regions in which the red-coloured region is nucleophile and electron rich, the blue colour indicates the electron-poor electrophile region and the white region indicates neutral atoms. These sites provide information about where the intermolecular inter­actions are involved within the mol­ecule (Gunasekaran *et al.*, 2008 ▸). The reactive sites are found near the carbonyl group: the region is represented in red and possesses the most negative potential spots. This nucleophile site (negative potential value of −0.04713 a.u.) is distributed around the oxygen atom due to the inter­molecular C—H⋯O inter­actions; in the mol­ecular structure it indicates the strong­est repulsion site (electrophilic attack), whereas the strongest attraction regions (nucleophilic attack) portrayed by the blue spots are localized on the hydrogen atoms.

## Database survey   

A search of the Cambridge Structural Database (Version 5.39, last update November 2017; Groom *et al.*, 2016 ▸) revealed one closely related compound that differs in the halogen substitution attached to the aldehyde ring, namely 3-(3-bromo­phen­yl)-1-(4-bromo­phen­yl)-prop-2-en-1-one (Teh *et al.*, 2006 ▸). Other related compounds, which differ in the halogen substitution at the *para*-position of the aldehyde ring include (2*E*)-1-(4-bromo­phen­yl)-3-(4-fluoro­phen­yl)prop-2-en-1-one (Dut­kiewicz *et al.*, 2010 ▸), 1-(4-bromo­phen­yl)-3-(4-chloro­phen­yl)prop-2-en-1-one (Yang *et al.*, 2006 ▸), 1,3-bis­(4-bromo­phen­yl)prop-2-en-1-one (Ng *et al.*, 2006 ▸), (*E*)-1-(4-bromo­phen­yl)-3-(4-iodo­ophen­yl)prop-2-en-1-one (Zainuri *et al.*, 2017 ▸) and (*E*)-3-(4-bromo­phen­yl)-1-(4-fluoro­phen­yl)prop-2-en-1-one (Zaini *et al.*, 2018 ▸).

## Synthesis and crystallization   

The title compound was prepared by a standard Claisen–Schmidt condensation reaction at room temperature. A mixture of 4-bromo­aceto­phenone (0.5 mmol) and 3-fluoro­benzaldehyde (0.5 mmol) was dissolved in methanol (20 ml) and the solution stirred continuously. A catalytic amount of NaOH (5 ml, 20%) was added to the solution dropwise until a precipitate formed and the reaction was stirred continuously for about 5 h. After stirring, the solution was poured into 60 ml of ice-cold distilled water. The resultant crude product was filtered and washed successively with distilled water until the filtrate turned colourless. The dried precipitate was further recrystallized to obtain the desired chalcone. Crystals suitable for X-ray diffraction analysis were formed by slow evaporation of an acetone solution.

## Refinement   

Details of the crystal data collection and structure refinement are summarized in Table 2 ▸. All C-bound H atoms were positioned geometrically (C—H = 0.930 Å) and refined using a riding model with *U*_iso_(H) = 1.2*U*_eq_(C). One outlier (3
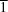
1) was omitted in the last cycles of refinement.

## Supplementary Material

Crystal structure: contains datablock(s) I. DOI: 10.1107/S2056989018017371/rz5249sup1.cif

Structure factors: contains datablock(s) I. DOI: 10.1107/S2056989018017371/rz5249Isup2.hkl

Comparison of bond lengths and angles between experimental and theoretical studies. DOI: 10.1107/S2056989018017371/rz5249sup3.docx

Supporting information file. DOI: 10.1107/S2056989018017371/rz5249Isup4.cml

CCDC reference: 1878940

Additional supporting information:  crystallographic information; 3D view; checkCIF report

## Figures and Tables

**Table 1:** Hydrogen-bond geometry (Å, °)

*D*—H⋯*A*	*D*—H	H⋯*A*	*D*⋯*A*	*D*—H⋯*A*
C13*A*—H13*A*⋯O1*B*	0.93	2.52	3.427 (4)	165
C9*A*—H9*AA*⋯O1*A* ^i^	0.93	2.52	3.362 (4)	151
C11*A*—H11*A*⋯O1*A* ^i^	0.93	2.45	3.294 (4)	151
C15*B*—H15*B*⋯O1*B* ^ii^	0.93	2.50	3.377 (4)	157

Symmetry codes: (i) 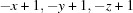; (ii) 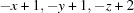.

**Table 2:** Experimental details

Crystal data	
Chemical formula	C_15_H_10_BrFO
*M*_r_	305.14
Crystal system, space group	Triclinic, *P* 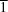
Temperature (K)	296
*a*, *b*, *c* (Å)	6.0090 (4), 10.8695 (7), 20.5616 (12)
α, β, γ (°)	102.803 (1), 96.938 (1), 97.276 (1)
*V* (Å^3^)	1283.57 (14)
*Z*	4
Radiation type	Mo *K*α
μ (mm^−1^)	3.20
Crystal size (mm)	0.56 × 0.39 × 0.29

Data collection	
Diffractometer	Bruker SMART APEXII Duo CCD area-detector
Absorption correction	Multi-scan (*SADABS*; Bruker, 2009 ▸)
*T*_min_, *T*_max_	0.267, 0.455
No. of measured, independent and observed [*I* > 2σ(*I*)] reflections	27389, 7437, 4832
*R*_int_	0.037
(sin θ/λ)_max_ (Å^−1^)	0.703

Refinement	
*R*[*F* ^2^ > 2σ(*F* ^2^)], *wR*(*F* ^2^), *S*	0.048, 0.158, 1.04
No. of reflections	7437
No. of parameters	325
H-atom treatment	H atoms treated by a mixture of independent and constrained refinement
Δρ_max_, Δρ_min_ (e Å^−3^)	0.85, −1.35

Computer programs: *APEX2* and *SAINT* (Bruker, 2009 ▸) and *SHELXTL* (Sheldrick, 2008 ▸) and *PLATON* (Spek, 2009 ▸).

## supplementary crystallographic information

### Crystal data

**Table:**

C_15_H_10_BrFO	*Z* = 4
*M**_r_* = 305.14	*F*(000) = 608
Triclinic, *P*1	*D*_x_ = 1.579 Mg m^−^^3^
Hall symbol: -P 1	Mo *K*α radiation, λ = 0.71073 Å
*a* = 6.0090 (4) Å	Cell parameters from 8958 reflections
*b* = 10.8695 (7) Å	θ = 2.5–28.0°
*c* = 20.5616 (12) Å	µ = 3.20 mm^−^^1^
α = 102.803 (1)°	*T* = 296 K
β = 96.938 (1)°	Plate, yellow
γ = 97.276 (1)°	0.56 × 0.39 × 0.29 mm
*V* = 1283.57 (14) Å^3^	

### Data collection

**Table:**

Bruker SMART APEXII Duo CCD area-detector diffractometer	7437 independent reflections
Radiation source: fine-focus sealed tube	4832 reflections with *I* > 2σ(*I*)
Graphite monochromator	*R*_int_ = 0.037
φ and ω scans	θ_max_ = 30.0°, θ_min_ = 2.0°
Absorption correction: multi-scan (*SADABS*; Bruker, 2009)	*h* = −8→8
*T*_min_ = 0.267, *T*_max_ = 0.455	*k* = −15→15
27389 measured reflections	*l* = −28→28

### Refinement

**Table:**

Refinement on *F*^2^	Primary atom site location: structure-invariant direct methods
Least-squares matrix: full	Secondary atom site location: difference Fourier map
*R*[*F*^2^ > 2σ(*F*^2^)] = 0.048	Hydrogen site location: inferred from neighbouring sites
*wR*(*F*^2^) = 0.158	H atoms treated by a mixture of independent and constrained refinement
*S* = 1.04	*w* = 1/[σ^2^(*F*_o_^2^) + (0.0792*P*)^2^ + 0.4733*P*] where *P* = (*F*_o_^2^ + 2*F*_c_^2^)/3
7437 reflections	(Δ/σ)_max_ = 0.001
325 parameters	Δρ_max_ = 0.85 e Å^−^^3^
0 restraints	Δρ_min_ = −1.35 e Å^−^^3^

### Special details

**Table:**

Geometry. All esds (except the esd in the dihedral angle between two l.s. planes) are estimated using the full covariance matrix. The cell esds are taken into account individually in the estimation of esds in distances, angles and torsion angles; correlations between esds in cell parameters are only used when they are defined by crystal symmetry. An approximate (isotropic) treatment of cell esds is used for estimating esds involving l.s. planes.
Refinement. Refinement of F^2^ against ALL reflections. The weighted R-factor wR and goodness of fit S are based on F^2^, conventional R-factors R are based on F, with F set to zero for negative F^2^. The threshold expression of F^2^ > 2sigma(F^2^) is used only for calculating R-factors(gt) etc. and is not relevant to the choice of reflections for refinement. R-factors based on F^2^ are statistically about twice as large as those based on F, and R- factors based on ALL data will be even larger.

### Fractional atomic coordinates and isotropic or equivalent isotropic displacement parameters (Å^2^)

**Table:**

	*x*	*y*	*z*	*U*_iso_*/*U*_eq_	
Br1A	−0.36851 (7)	1.01488 (4)	0.369778 (19)	0.07958 (15)	
F1A	0.5304 (4)	0.3263 (2)	0.74576 (11)	0.0894 (7)	
O1A	0.3367 (4)	0.6239 (3)	0.46486 (13)	0.0805 (8)	
C1A	−0.1316 (5)	0.7958 (3)	0.49478 (14)	0.0533 (6)	
H1AA	−0.1703	0.7725	0.5333	0.064*	
C2A	−0.2576 (5)	0.8732 (3)	0.46588 (16)	0.0595 (7)	
H2AA	−0.3807	0.9017	0.4846	0.071*	
C3A	−0.1989 (5)	0.9074 (3)	0.40938 (15)	0.0564 (7)	
C4A	−0.0175 (6)	0.8661 (3)	0.38068 (16)	0.0632 (8)	
H4AA	0.0205	0.8903	0.3423	0.076*	
C5A	0.1071 (5)	0.7882 (3)	0.40975 (15)	0.0583 (7)	
H5AA	0.2293	0.7596	0.3906	0.070*	
C6A	0.0523 (4)	0.7524 (2)	0.46713 (13)	0.0462 (5)	
C7A	0.1930 (5)	0.6668 (3)	0.49525 (14)	0.0526 (6)	
C8A	0.1559 (5)	0.6354 (3)	0.55997 (14)	0.0513 (6)	
H8AA	0.0430	0.6682	0.5824	0.062*	
C9A	0.2801 (5)	0.5616 (3)	0.58648 (13)	0.0492 (6)	
H9AA	0.3932	0.5324	0.5628	0.059*	
C10A	0.2592 (5)	0.5208 (2)	0.64885 (12)	0.0457 (5)	
C11A	0.4081 (5)	0.4430 (3)	0.66883 (14)	0.0531 (6)	
H11A	0.5195	0.4181	0.6433	0.064*	
C12A	0.3866 (6)	0.4042 (3)	0.72699 (15)	0.0606 (7)	
C13A	0.2265 (6)	0.4381 (3)	0.76688 (15)	0.0685 (9)	
H13A	0.2171	0.4102	0.8062	0.082*	
C14A	0.0807 (7)	0.5147 (4)	0.74667 (17)	0.0750 (9)	
H14A	−0.0297	0.5390	0.7727	0.090*	
C15A	0.0953 (6)	0.5562 (3)	0.68819 (15)	0.0637 (8)	
H15A	−0.0050	0.6080	0.6752	0.076*	
Br1B	−0.33082 (7)	−0.20429 (4)	0.72238 (2)	0.09061 (17)	
F1B	1.2882 (4)	0.0939 (2)	1.06936 (14)	0.1021 (8)	
O1B	0.3006 (4)	0.34757 (19)	0.91642 (11)	0.0632 (5)	
C1B	−0.0157 (5)	0.1510 (3)	0.83454 (15)	0.0538 (6)	
H1BA	−0.0513	0.2333	0.8440	0.065*	
C2B	−0.1694 (5)	0.0535 (3)	0.79170 (15)	0.0587 (7)	
H2BA	−0.3074	0.0694	0.7722	0.070*	
C3B	−0.1153 (5)	−0.0677 (3)	0.77835 (16)	0.0590 (7)	
C4B	0.0883 (6)	−0.0930 (3)	0.80586 (19)	0.0730 (9)	
H4BA	0.1231	−0.1754	0.7959	0.088*	
C5B	0.2415 (5)	0.0063 (3)	0.84882 (17)	0.0646 (8)	
H5BA	0.3799	−0.0101	0.8677	0.077*	
C6B	0.1921 (5)	0.1289 (3)	0.86397 (13)	0.0484 (6)	
C7B	0.3501 (5)	0.2405 (2)	0.90990 (13)	0.0483 (6)	
C8B	0.5623 (5)	0.2192 (3)	0.94716 (14)	0.0522 (6)	
H8BA	0.6019	0.1378	0.9396	0.063*	
C9B	0.6961 (5)	0.3151 (3)	0.99126 (13)	0.0481 (5)	
H9BA	0.6479	0.3943	0.9971	0.058*	
C10B	0.9115 (5)	0.3113 (2)	1.03206 (12)	0.0458 (5)	
C11B	0.9974 (5)	0.1983 (3)	1.03227 (15)	0.0574 (7)	
H11B	0.9164	0.1202	1.0071	0.069*	
C12B	1.2032 (6)	0.2045 (3)	1.07027 (16)	0.0623 (7)	
C13B	1.3312 (5)	0.3157 (3)	1.10868 (15)	0.0611 (7)	
H13B	1.4703	0.3159	1.1341	0.073*	
C14B	1.2459 (6)	0.4271 (3)	1.10829 (14)	0.0607 (7)	
H14B	1.3291	0.5046	1.1335	0.073*	
C15B	1.0377 (5)	0.4254 (3)	1.07085 (13)	0.0527 (6)	
H15B	0.9816	0.5016	1.0717	0.063*	

### Atomic displacement parameters (Å^2^)

**Table:**

	*U*^11^	*U*^22^	*U*^33^	*U*^12^	*U*^13^	*U*^23^
Br1A	0.0856 (3)	0.0670 (2)	0.0818 (3)	0.01918 (18)	−0.02313 (19)	0.02390 (17)
F1A	0.1164 (18)	0.0866 (14)	0.0753 (13)	0.0378 (13)	−0.0027 (12)	0.0375 (11)
O1A	0.0840 (17)	0.1058 (19)	0.0857 (16)	0.0548 (15)	0.0438 (14)	0.0557 (15)
C1A	0.0548 (15)	0.0585 (16)	0.0497 (14)	0.0147 (12)	0.0070 (12)	0.0166 (12)
C2A	0.0569 (16)	0.0606 (17)	0.0612 (16)	0.0184 (13)	0.0026 (13)	0.0134 (13)
C3A	0.0599 (16)	0.0471 (14)	0.0552 (15)	0.0058 (12)	−0.0141 (13)	0.0115 (11)
C4A	0.073 (2)	0.0651 (18)	0.0542 (16)	0.0068 (15)	0.0018 (14)	0.0268 (14)
C5A	0.0599 (17)	0.0622 (17)	0.0578 (16)	0.0161 (13)	0.0103 (13)	0.0206 (13)
C6A	0.0467 (13)	0.0440 (12)	0.0471 (13)	0.0050 (10)	0.0029 (10)	0.0128 (10)
C7A	0.0535 (15)	0.0521 (14)	0.0570 (15)	0.0134 (12)	0.0103 (12)	0.0191 (12)
C8A	0.0537 (15)	0.0519 (14)	0.0521 (14)	0.0143 (12)	0.0119 (12)	0.0153 (11)
C9A	0.0504 (14)	0.0487 (13)	0.0495 (13)	0.0093 (11)	0.0076 (11)	0.0129 (11)
C10A	0.0509 (14)	0.0421 (12)	0.0417 (12)	0.0054 (10)	0.0032 (10)	0.0083 (10)
C11A	0.0605 (16)	0.0479 (13)	0.0482 (14)	0.0101 (12)	0.0009 (12)	0.0090 (11)
C12A	0.075 (2)	0.0516 (15)	0.0514 (15)	0.0051 (13)	−0.0077 (14)	0.0159 (12)
C13A	0.083 (2)	0.078 (2)	0.0440 (15)	0.0016 (17)	0.0046 (15)	0.0221 (14)
C14A	0.077 (2)	0.098 (3)	0.0557 (17)	0.021 (2)	0.0217 (16)	0.0213 (18)
C15A	0.071 (2)	0.0728 (19)	0.0533 (16)	0.0248 (16)	0.0120 (14)	0.0185 (14)
Br1B	0.0727 (3)	0.0615 (2)	0.1139 (3)	0.01018 (17)	−0.0328 (2)	−0.00307 (19)
F1B	0.0907 (16)	0.0653 (13)	0.140 (2)	0.0298 (11)	−0.0343 (15)	0.0209 (13)
O1B	0.0684 (13)	0.0461 (10)	0.0725 (13)	0.0170 (9)	−0.0053 (10)	0.0128 (9)
C1B	0.0542 (16)	0.0488 (13)	0.0599 (15)	0.0171 (11)	0.0020 (12)	0.0147 (12)
C2B	0.0498 (15)	0.0613 (17)	0.0632 (17)	0.0151 (13)	−0.0054 (13)	0.0153 (13)
C3B	0.0537 (16)	0.0529 (15)	0.0638 (16)	0.0070 (12)	−0.0068 (13)	0.0091 (13)
C4B	0.0655 (19)	0.0492 (16)	0.093 (2)	0.0170 (14)	−0.0145 (17)	0.0023 (15)
C5B	0.0523 (16)	0.0541 (16)	0.081 (2)	0.0168 (13)	−0.0117 (14)	0.0102 (14)
C6B	0.0473 (13)	0.0519 (14)	0.0472 (13)	0.0113 (11)	0.0024 (11)	0.0151 (11)
C7B	0.0513 (14)	0.0462 (13)	0.0483 (13)	0.0105 (11)	0.0034 (11)	0.0138 (10)
C8B	0.0528 (15)	0.0472 (13)	0.0567 (15)	0.0134 (11)	0.0017 (12)	0.0134 (11)
C9B	0.0520 (14)	0.0462 (13)	0.0477 (13)	0.0114 (11)	0.0055 (11)	0.0135 (10)
C10B	0.0506 (14)	0.0462 (13)	0.0410 (12)	0.0097 (10)	0.0056 (10)	0.0112 (10)
C11B	0.0600 (16)	0.0450 (13)	0.0626 (16)	0.0068 (12)	−0.0056 (13)	0.0125 (12)
C12B	0.0648 (18)	0.0560 (16)	0.0659 (17)	0.0164 (14)	−0.0031 (14)	0.0176 (14)
C13B	0.0552 (16)	0.0719 (19)	0.0547 (15)	0.0077 (14)	−0.0022 (13)	0.0190 (14)
C14B	0.0633 (18)	0.0603 (17)	0.0484 (14)	0.0039 (13)	−0.0039 (13)	0.0017 (12)
C15B	0.0635 (17)	0.0485 (14)	0.0438 (13)	0.0121 (12)	0.0054 (12)	0.0060 (11)

### Geometric parameters (Å, º)

**Table:**

Br1A—C3A	1.894 (3)	Br1B—C3B	1.898 (3)
F1A—C12A	1.365 (4)	F1B—C12B	1.362 (4)
O1A—C7A	1.207 (4)	O1B—C7B	1.221 (3)
C1A—C2A	1.381 (4)	C1B—C2B	1.376 (4)
C1A—C6A	1.390 (4)	C1B—C6B	1.393 (4)
C1A—H1AA	0.9300	C1B—H1BA	0.9300
C2A—C3A	1.367 (5)	C2B—C3B	1.374 (4)
C2A—H2AA	0.9300	C2B—H2BA	0.9300
C3A—C4A	1.378 (5)	C3B—C4B	1.371 (4)
C4A—C5A	1.383 (4)	C4B—C5B	1.386 (4)
C4A—H4AA	0.9300	C4B—H4BA	0.9300
C5A—C6A	1.385 (4)	C5B—C6B	1.379 (4)
C5A—H5AA	0.9300	C5B—H5BA	0.9300
C6A—C7A	1.494 (4)	C6B—C7B	1.499 (4)
C7A—C8A	1.481 (4)	C7B—C8B	1.477 (4)
C8A—C9A	1.321 (4)	C8B—C9B	1.322 (4)
C8A—H8AA	0.9300	C8B—H8BA	0.9300
C9A—C10A	1.461 (4)	C9B—C10B	1.465 (4)
C9A—H9AA	0.9300	C9B—H9BA	0.9300
C10A—C15A	1.387 (4)	C10B—C15B	1.388 (4)
C10A—C11A	1.393 (4)	C10B—C11B	1.393 (4)
C11A—C12A	1.368 (4)	C11B—C12B	1.366 (4)
C11A—H11A	0.9300	C11B—H11B	0.9300
C12A—C13A	1.371 (5)	C12B—C13B	1.368 (5)
C13A—C14A	1.373 (5)	C13B—C14B	1.375 (5)
C13A—H13A	0.9300	C13B—H13B	0.9300
C14A—C15A	1.383 (5)	C14B—C15B	1.383 (4)
C14A—H14A	0.9300	C14B—H14B	0.9300
C15A—H15A	0.9300	C15B—H15B	0.9300
			
C2A—C1A—C6A	121.0 (3)	C2B—C1B—C6B	121.4 (3)
C2A—C1A—H1AA	119.5	C2B—C1B—H1BA	119.3
C6A—C1A—H1AA	119.5	C6B—C1B—H1BA	119.3
C3A—C2A—C1A	119.1 (3)	C3B—C2B—C1B	118.8 (3)
C3A—C2A—H2AA	120.4	C3B—C2B—H2BA	120.6
C1A—C2A—H2AA	120.4	C1B—C2B—H2BA	120.6
C2A—C3A—C4A	121.4 (3)	C4B—C3B—C2B	121.6 (3)
C2A—C3A—Br1A	119.5 (2)	C4B—C3B—Br1B	119.2 (2)
C4A—C3A—Br1A	119.0 (2)	C2B—C3B—Br1B	119.2 (2)
C3A—C4A—C5A	119.1 (3)	C3B—C4B—C5B	118.9 (3)
C3A—C4A—H4AA	120.5	C3B—C4B—H4BA	120.5
C5A—C4A—H4AA	120.5	C5B—C4B—H4BA	120.5
C4A—C5A—C6A	120.8 (3)	C6B—C5B—C4B	121.1 (3)
C4A—C5A—H5AA	119.6	C6B—C5B—H5BA	119.5
C6A—C5A—H5AA	119.6	C4B—C5B—H5BA	119.5
C5A—C6A—C1A	118.6 (3)	C5B—C6B—C1B	118.2 (3)
C5A—C6A—C7A	117.8 (2)	C5B—C6B—C7B	123.8 (2)
C1A—C6A—C7A	123.6 (2)	C1B—C6B—C7B	118.0 (2)
O1A—C7A—C8A	120.4 (3)	O1B—C7B—C8B	120.9 (3)
O1A—C7A—C6A	119.5 (2)	O1B—C7B—C6B	119.5 (2)
C8A—C7A—C6A	120.0 (2)	C8B—C7B—C6B	119.6 (2)
C9A—C8A—C7A	121.3 (3)	C9B—C8B—C7B	120.4 (2)
C9A—C8A—H8AA	119.4	C9B—C8B—H8BA	119.8
C7A—C8A—H8AA	119.4	C7B—C8B—H8BA	119.8
C8A—C9A—C10A	127.1 (3)	C8B—C9B—C10B	127.6 (2)
C8A—C9A—H9AA	116.4	C8B—C9B—H9BA	116.2
C10A—C9A—H9AA	116.4	C10B—C9B—H9BA	116.2
C15A—C10A—C11A	119.3 (2)	C15B—C10B—C11B	118.6 (3)
C15A—C10A—C9A	122.1 (3)	C15B—C10B—C9B	118.7 (2)
C11A—C10A—C9A	118.6 (2)	C11B—C10B—C9B	122.7 (2)
C12A—C11A—C10A	118.4 (3)	C12B—C11B—C10B	118.6 (3)
C12A—C11A—H11A	120.8	C12B—C11B—H11B	120.7
C10A—C11A—H11A	120.8	C10B—C11B—H11B	120.7
F1A—C12A—C11A	118.2 (3)	F1B—C12B—C11B	118.3 (3)
F1A—C12A—C13A	118.3 (3)	F1B—C12B—C13B	117.7 (3)
C11A—C12A—C13A	123.5 (3)	C11B—C12B—C13B	123.9 (3)
C12A—C13A—C14A	117.6 (3)	C12B—C13B—C14B	117.3 (3)
C12A—C13A—H13A	121.2	C12B—C13B—H13B	121.3
C14A—C13A—H13A	121.2	C14B—C13B—H13B	121.3
C13A—C14A—C15A	121.1 (3)	C13B—C14B—C15B	120.8 (3)
C13A—C14A—H14A	119.5	C13B—C14B—H14B	119.6
C15A—C14A—H14A	119.5	C15B—C14B—H14B	119.6
C14A—C15A—C10A	120.1 (3)	C14B—C15B—C10B	120.8 (3)
C14A—C15A—H15A	119.9	C14B—C15B—H15B	119.6
C10A—C15A—H15A	119.9	C10B—C15B—H15B	119.6
			
C6A—C1A—C2A—C3A	−0.2 (5)	C6B—C1B—C2B—C3B	0.3 (5)
C1A—C2A—C3A—C4A	0.2 (5)	C1B—C2B—C3B—C4B	−0.9 (5)
C1A—C2A—C3A—Br1A	−179.4 (2)	C1B—C2B—C3B—Br1B	177.4 (2)
C2A—C3A—C4A—C5A	0.1 (5)	C2B—C3B—C4B—C5B	0.8 (6)
Br1A—C3A—C4A—C5A	179.6 (2)	Br1B—C3B—C4B—C5B	−177.5 (3)
C3A—C4A—C5A—C6A	−0.3 (5)	C3B—C4B—C5B—C6B	−0.1 (6)
C4A—C5A—C6A—C1A	0.3 (5)	C4B—C5B—C6B—C1B	−0.5 (5)
C4A—C5A—C6A—C7A	179.3 (3)	C4B—C5B—C6B—C7B	−179.9 (3)
C2A—C1A—C6A—C5A	0.0 (4)	C2B—C1B—C6B—C5B	0.4 (4)
C2A—C1A—C6A—C7A	−179.0 (3)	C2B—C1B—C6B—C7B	179.8 (3)
C5A—C6A—C7A—O1A	−5.7 (4)	C5B—C6B—C7B—O1B	174.3 (3)
C1A—C6A—C7A—O1A	173.3 (3)	C1B—C6B—C7B—O1B	−5.2 (4)
C5A—C6A—C7A—C8A	174.3 (3)	C5B—C6B—C7B—C8B	−6.3 (4)
C1A—C6A—C7A—C8A	−6.8 (4)	C1B—C6B—C7B—C8B	174.2 (3)
O1A—C7A—C8A—C9A	1.0 (5)	O1B—C7B—C8B—C9B	3.9 (4)
C6A—C7A—C8A—C9A	−179.0 (3)	C6B—C7B—C8B—C9B	−175.5 (3)
C7A—C8A—C9A—C10A	−178.7 (3)	C7B—C8B—C9B—C10B	−179.7 (3)
C8A—C9A—C10A—C15A	0.9 (5)	C8B—C9B—C10B—C15B	173.9 (3)
C8A—C9A—C10A—C11A	−179.6 (3)	C8B—C9B—C10B—C11B	−4.5 (5)
C15A—C10A—C11A—C12A	0.0 (4)	C15B—C10B—C11B—C12B	−0.5 (4)
C9A—C10A—C11A—C12A	−179.4 (3)	C9B—C10B—C11B—C12B	177.9 (3)
C10A—C11A—C12A—F1A	178.9 (3)	C10B—C11B—C12B—F1B	−178.9 (3)
C10A—C11A—C12A—C13A	−0.2 (5)	C10B—C11B—C12B—C13B	0.2 (5)
F1A—C12A—C13A—C14A	−178.9 (3)	F1B—C12B—C13B—C14B	178.8 (3)
C11A—C12A—C13A—C14A	0.3 (5)	C11B—C12B—C13B—C14B	−0.3 (5)
C12A—C13A—C14A—C15A	−0.1 (6)	C12B—C13B—C14B—C15B	0.6 (5)
C13A—C14A—C15A—C10A	−0.1 (6)	C13B—C14B—C15B—C10B	−0.9 (5)
C11A—C10A—C15A—C14A	0.1 (5)	C11B—C10B—C15B—C14B	0.8 (4)
C9A—C10A—C15A—C14A	179.6 (3)	C9B—C10B—C15B—C14B	−177.7 (3)

### Hydrogen-bond geometry (Å, º)

**Table:**

*D*—H···*A*	*D*—H	H···*A*	*D*···*A*	*D*—H···*A*
C13*A*—H13*A*···O1*B*	0.93	2.52	3.427 (4)	165
C9*A*—H9*AA*···O1*A*^i^	0.93	2.52	3.362 (4)	151
C11*A*—H11*A*···O1*A*^i^	0.93	2.45	3.294 (4)	151
C15*B*—H15*B*···O1*B*^ii^	0.93	2.50	3.377 (4)	157

Symmetry codes: (i) −*x*+1, −*y*+1, −*z*+1; (ii) −*x*+1, −*y*+1, −*z*+2.
