# Supplementary material for: Crystal structure, Hirshfeld surface analysis and DFT studies of (E)-1-(4-bromo­phen­yl)-3-(3-fluoro­phen­yl)prop-2-en-1-one
Source: Acta Crystallogr E Crystallogr Commun. 2019 Jan 1;75(Pt 1):58–63. doi: 10.1107/S2056989018017371 (PMC6323881; doi:10.1107/S2056989018017371)
Supplement: Supplementary file 3 [file e-75-00058-sup3.docx]

Supplementary information

Table S1: Comparison of bond lengths and angles between experimental and theoretical studies

| **Bond Distance (Å)** | | | |
| --- | --- | --- | --- |
| Atoms | Experimental | | DFT B3LYP/6-311 G++(d,p) |
|  | A | B |  |
| O1 - C7 | 1.207 (4) | 1.221 (3) | 1.22340 |
| C6 - C7 | 1.494 (4) | 1.499 (4) | 1.50193 |
| C7 - C8 | 1.481 (4) | 1.477 (4) | 1.48483 |
| C8 - C9 | 1.321 (4) | 1.322 (4) | 1.34477 |
| C9 - C10 | 1.461 (4) | 1.465 (4) | 1.46252 |
| C3 - Br | 1.894 (3) | 1.898 (3) | 1.91373 |
| C12 - F1 | 1.365 (4) | 1.362 (4) | 1.35417 |

| Bond Angle (°) | | | |
| --- | --- | --- | --- |
| Atoms | Experimental | | DFT B3LYP/6-311 G++(d,p) |
|  | A | B |  |
| O1 - C7 - C8 | 120.4 (3) | 120.9 (3) | 121.35757 |
| O1 - C7 - C6 | 119.5 (2) | 119.5 (2) | 119.79222 |
| C6 - C7 - C8 | 120.0 (2) | 119.6 (2) | 118.84446 |
| C7 - C8 - C9 | 121.3 (3) | 120.4 (2) | 120.27545 |
| C8 - C9 - C10 | 127.1 (3) | 127.6 (2) | 127.73159 |
| C9 - C10 - C11 | 118.6 (2) | 122.7 (2) | 118.06387 |
| C9 - C10 - C15 | 122.1 (3) | 118.7 (2) | 123.44812 |
| C1 - C6 - C7 | 123.6 (2) | 118.0 (2) | 123.62246 |
| C5 - C6 - C7 | 117.8 (2) | 123.8 (2) | 117.76930 |
| C4 - C3 - Br1 | 119.0 (2) | 119.2 (2) | 119.38678 |
| C2 - C3 - Br1 | 119.5 (2) | 119.2 (2) | 119.39510 |
| C11 - C12- F1 | 118.2 (3) | 118.3 (3) | 118.62291 |
| C13 - C12 - F1 | 118.3 (3) | 117.7 (3) | 118.85658 |

| Torsion Angle (°) | | | |
| --- | --- | --- | --- |
| Atoms | Experimental | | DFT B3LYP/6-311 G++(d,p) |
|  | A | B |  |
| C5 - C6 - C7 - O1 | −5.7 (4) | 174.3 (3) | -13.42528 |
| C1 - C6 - C7 - O1 | 173.3 (3) | −5.2 (4) | 165.23589 |
| O1 - C7 - C8 - C9 | 1.0 (5) | 3.9 (4) | -5.02400 |
| C6 - C7 - C8 - C9 | −179.0 (3) | −175.5 (3) | 175.85013 |
| C7 - C8 - C9 - C10 | −178.7 (3) | −179.7 (3) | 178.96464 |
| C8 - C9 - C10 - C15 | 0.9 (5) | 173.9 (3) | -2.12537 |
| C8 - C9 - C10 - C11 | −179.6 (3) | −4.5 (5) | 177.97357 |
